# Kinesiophobia, limits of stability, and functional balance assessment in geriatric patients with chronic low back pain and osteoporosis: a comprehensive study

**DOI:** 10.3389/fneur.2024.1354444

**Published:** 2024-02-13

**Authors:** Mastour Saeed Alshahrani, Ravi Shankar Reddy

**Affiliations:** Program of Physical Therapy, Department of Medical Rehabilitation Sciences, College of Applied Medical Sciences, King Khalid University, Abha, Saudi Arabia

**Keywords:** chronic low back pain, osteoporosis, Kinesiophobia, limits of stability, functional balance, geriatric patients

## Abstract

**Background:**

The significance of studying Kinesiophobia, Limits of Stability (LOS), and functional balance in geriatric patients with CLBP and osteoporosis lies in their profound impact on rehabilitation outcomes and fall risk, ultimately affecting patients’ quality of life. This study aimed to examine LOS and functional balance in the geriatric population concurrently experiencing Chronic Low Back Pain (CLBP) and osteoporosis, in comparison to age-matched healthy controls; to assess the correlations between Kinesiophobia, LOS, and functional balance assessments; and to evaluate the mediating influence of Kinesiophobia on the association between LOS and functional balance tests.

**Methods:**

This cross-sectional study included a total of 86 participants in each group. Kinesiophobia was assessed using the Tampa Scale of Kinesiophobia (TSK). LOS variables were evaluated with a computerized Iso-free platform in eight different directions. Functional balance was measured using the Timed Up and Go (TUG) test and the Berg Balance Scale (BBS).

**Results:**

Patients with CLBP and osteoporosis showed significantly lower LOS percentages (45.78 ± 6.92) and impaired Functional Balance, reflected in a TUG Score (10.45 ± 2.23), compared to asymptomatic controls (LOS: 76.95 ± 8.21; TUG: 8.73 ± 1.90). Kinesiophobia showed a significant moderate negative correlation with LOS, indicated by r = −0.362 (*p* < 0.01). Additionally, Kinesiophobia was found to correlate with functional balance tests. Specifically, there was a moderate positive correlation with the TUG Score (r = 0.322, *p* < 0.01), indicating that higher Kinesiophobia is associated with slower TUG performance. Conversely, a stronger moderate negative correlation was observed with the Berg Balance Scale (BBS) Score (r = −0.436, *p* < 0.001), suggesting that increased Kinesiophobia is associated with lower BBS scores, indicating poorer balance performance. Mediation analysis revealed that Kinesiophobia significantly influences LOS and Functional Balance. For LOS and the TUG score, Kinesiophobia showed a direct effect (*B* = 0.24), an indirect effect (*B* = 0.09), and a total effect (*B* = 0.13). Similarly, for LOS and the BBS score, the direct effect of Kinesiophobia was *B* = 0.38, with an indirect effect of *B* = 0.10 and a total effect of *B* = 0.20.

**Conclusion:**

This study underscores the substantial impact of Kinesiophobia on both stability and functional balance in individuals coping with CLBP and osteoporosis. The findings emphasize the clinical relevance of addressing Kinesiophobia as a potential target for interventions aimed at improving LOS and functional balance in this specific patient population.

## 1 Introduction

Chronic Low Back Pain (CLBP) represents a pervasive and incapacitating musculoskeletal disorder with a global prevalence, impacting a substantial populace of individuals across the world (1). The global prevalence of lower back pain (LBP) is substantial, with an estimated 619 million people affected, making it the leading cause of disability worldwide (2). It is characterized by persistent pain and discomfort in the lumbar region, often leading to impaired physical function and reduced quality of life (3). CLBP may arise from diverse etiologies, encompassing degenerative disc disease, intervertebral disc herniation, spinal stenosis, or musculo-ligamentous strains (4). One particularly challenging aspect of managing CLBP is the presence of comorbidities, such as osteoporosis, which can further exacerbate the pain and functional limitations experienced by affected individuals (5). Osteoporosis is a systemic skeletal disorder typified by a reduction in bone mass and density, predisposing individuals to an elevated susceptibility to fractures (6). Osteoporosis has a significant global impact, particularly among women, affecting approximately 200 million worldwide. This prevalence escalates with age, affecting one-tenth of women at age 60, one-fifth by age 70, two-fifths at age 80, and two-thirds by age 90 (7). The coexistence of CLBP and osteoporosis in the same individual can create a complex clinical picture, as both conditions can independently contribute to reduced physical function and mobility (8).

Postural control refers to the intricate process by which the human body maintains its balance and stability while assuming various positions and performing activities (9). It is a fundamental aspect of our daily lives, allowing us to sit, stand, walk, and engage in dynamic movements with ease and coordination (10). Achieving effective postural control involves the integration of sensory information from the visual, vestibular, and proprioceptive systems, which helps the brain make real-time adjustments to muscle contractions and joint movements (11). A well-functioning postural control system is crucial for preventing falls, supporting efficient movement, and reducing the risk of musculoskeletal problems (12). It is a key focus in various fields, including physical therapy, sports performance, and rehabilitation, as it plays a vital role in maintaining overall health and functionality (13).

Functional balance in geriatrics refers to the ability of older individuals to maintain stability and control their bodies during daily activities (14). As people age, various factors, such as muscle weakness, joint stiffness, reduced proprioception, and changes in sensory perception, can affect their balance and coordination (15). Functional balance is essential for older adults as it directly impacts their independence and quality of life (16). Maintaining good functional balance allows seniors to perform routine tasks like walking, getting up from a chair, and reaching for objects safely and efficiently (17). Moreover, it plays a crucial role in fall prevention, a significant concern in the aging population, as falls can lead to serious injuries and a decline in overall health (17).

Kinesiophobia, defined as an excessive and irrational fear of movement due to the perceived risk of pain or re-injury, significantly impacts the experience of chronic lower back pain (CLBP) (18). It often emerges as a protective response in individuals with CLBP, leading to avoidance of physical activities and a decline in functional capacity (18). The presence of osteoporosis can further exacerbate this fear due to concerns about bone fragility and potential fractures during movement (19). Therefore, comprehending the role of Kinesiophobia in the context of CLBP and osteoporosis is vital for devising effective rehabilitation strategies (18).

CLBP frequently coincides with Kinesiophobia, creating a cycle of pain avoidance and physical deconditioning (20). Exploring how Kinesiophobia might mediate the relationship between limits of stability (LOS) and functional balance provides critical insights into the mechanisms contributing to functional impairments in CLBP patients (21, 22). This understanding sheds light on the psychological factors influencing an individual’s ability to maintain postural stability and perform daily activities, ultimately impacting their quality of life (23). Moreover, by recognizing Kinesiophobia as a potential mediator, we open avenues for targeted interventions aimed at reducing fear-avoidance behaviors, enhancing postural control, and improving functional balance in this specific patient population (24). This knowledge has the potential to inform more effective rehabilitation strategies and enhance CLBP management, ultimately leading to improved patient outcomes and better daily functioning (24). Although previous research has separately examined the influence of Kinesiophobia on functional balance and the impact of osteoporosis on postural control (18, 25), there is a scarcity of studies comprehensively investigating how kinesiophobia mediates the relationship between Limits of Stability, a measure of postural control, and functional balance in individuals with CLBP and osteoporosis.

This study seeks to bridge existing gaps in the literature by pursuing the following primary objectives. This study aimed to examine LOS and Functional Balance in the geriatric population concurrently experiencing CLBP and osteoporosis, in comparison to age-matched healthy controls; to assess the correlations between Kinesiophobia, LOS, and functional balance assessments; and to evaluate the mediating influence of Kinesiophobia on the association between LOS and functional balance tests. We hypothesize that individuals with CLBP and osteoporosis will exhibit significantly lower LOS percentages and impaired functional balance compared to age-matched healthy controls. Furthermore, we anticipate that Kinesiophobia will mediate the association between Limits of Stability and functional balance in CLBP patients, highlighting its crucial role in shaping functional outcomes in this specific population.

## 2 Materials and methods

### 2.1 Study design, settings, and duration

This study utilized a prospective cross-sectional design to examine Kinesiophobia, LOS, and Functional Balance in individuals with co-occurring CLBP and osteoporosis, alongside age-matched healthy control subjects. The research was carried out within a clinical research environment in the field of physical therapy, with data collection spanning from March 2021 to December 2022 continuously without distinct phases or intervals. This study strictly adhered to ethical guidelines and received approval from the King Khalid University ethics committee. Prior to participation, subjects were provided comprehensive information about the study’s objectives, procedures, potential risks, and benefits. They were afforded ample time for questions, and clarifications, and provided written informed consent, signifying their comprehension and voluntary participation.

### 2.2 Participants

Participants were recruited from Osteoporosis and Physiotherapy clinics, KKU hospitals, and surrounding community centers through convenience sampling. The inclusion criteria for this study comprised individuals aged 55 years or older with a clinically confirmed diagnosis of CLBP persisting for a minimum of three months. Additionally, participants were required to demonstrate a T score of ≤ −2.5 at either the lumbar spine or hip, as determined by dual-energy X-ray absorptiometry (DXA), indicating the presence of osteoporosis. Our study employed dual-energy X-ray absorptiometry (DXA) scans for assessing bone density, a key factor in diagnosing osteoporosis. This method is recognized for its accuracy in evaluating bone strength and is integral in combination with the FRAX risk assessment tool for determining osteoporosis severity among participants. Pain severity was assessed using a standardized pain scale, with participants needing to report a pain score of 3 or higher on the Visual Analog Scale (VAS). The threshold of 3 or higher on the VAS was selected to ensure the inclusion of participants with clinically significant pain levels, crucial for examining the relationship between pain intensity, kinesiophobia, and rehabilitation outcomes. Eligible participants were those who expressed willingness to adhere to the study’s requirements, provided informed consent, and met stable medication use conditions. Conversely, exclusion criteria commonly included individuals presenting “red flag” symptoms indicative of potentially serious underlying conditions and pregnant individuals, ensuring the safety and homogeneity of the study cohort.

Inclusion and exclusion criteria were established to select age-matched asymptomatic subjects for this study. Inclusion criteria included individuals within the specified age range that closely matched the age of the study cohort consisting of patients with chronic low back pain and osteoporosis. Asymptomatic subjects were required to have no history of chronic low back pain or any musculoskeletal or neurological disorders that could potentially affect their balance or functional mobility. Exclusion criteria encompassed individuals with a history of acute or chronic back pain, osteoporosis, significant musculoskeletal or neurological conditions, recent injuries affecting balance, or any medical conditions or medications that could influence balance or functional capacity.

### 2.3 Sample size calculation

The sample size calculation for this study was performed using G*Power statistics software, to ensure adequate statistical power to detect meaningful effects. In line with previous research by Ucurum et al. (26), which reported an effect size of 0.4, this effect size was considered for the current study. To determine the appropriate sample size, a power analysis was conducted using a significance level (α) of 0.05 and a power (1-β) of 0.80. Based on these parameters and the chosen effect size, the sample size calculation yielded a required total sample size of 86 participants in each group.

### 2.4 Outcome measures

#### 2.4.1 Tampa scale of Kinesiophobia

Each participant was provided with a copy of the TSK questionnaire, which consists of 17 items related to concerns and fears regarding physical activity and pain. Participants were instructed to read each item carefully and indicate the extent to which they agreed with each statement on a 4-point Likert scale, with response options ranging from 1 (strongly disagree) to 4 (strongly agree). They were encouraged to respond honestly based on their feelings and experiences. Trained research personnel were available to address any questions or concerns participants had during the completion of the questionnaire. The research personnel administering the TSK were qualified professionals, trained in psychological assessments and experienced in clinical research, ensuring consistent and empathetic interaction with participants. Once all participants had finished filling out the TSK questionnaire, the responses were collected, and the scores for each participant were calculated by summing their responses to the 17 items (27). This total score represented the individual’s level of Kinesiophobia, with higher scores indicating higher levels of fear of movement. The TSK has demonstrated strong reliability and validity in assessing Kinesiophobia in patients with LBP, making it a valuable tool for accurately measuring fear-related avoidance behaviors and attitudes toward physical activity in this population (27).

#### 2.4.2 Limits of stability assessment

The Limits of Stability (LOS) assessment in this study adhered to stringent standardization procedures to ensure the accuracy and reliability of the collected data (28). Prior to commencing the assessment, participants were familiarized with the testing environment and procedures. They were provided with clear instructions on how to stand on the Iso-free stabilometric force platform and were encouraged to adopt a relaxed yet stable posture. The testing environment was carefully controlled to minimize external influences. Adequate lighting, temperature control, and a noise-free setting were maintained to create optimal conditions for the LOS assessment. Participants were instructed to wear comfortable clothing that did not restrict their movements, facilitating their ability to shift their Center of Gravity (COG) accurately.

**Figure 1:**
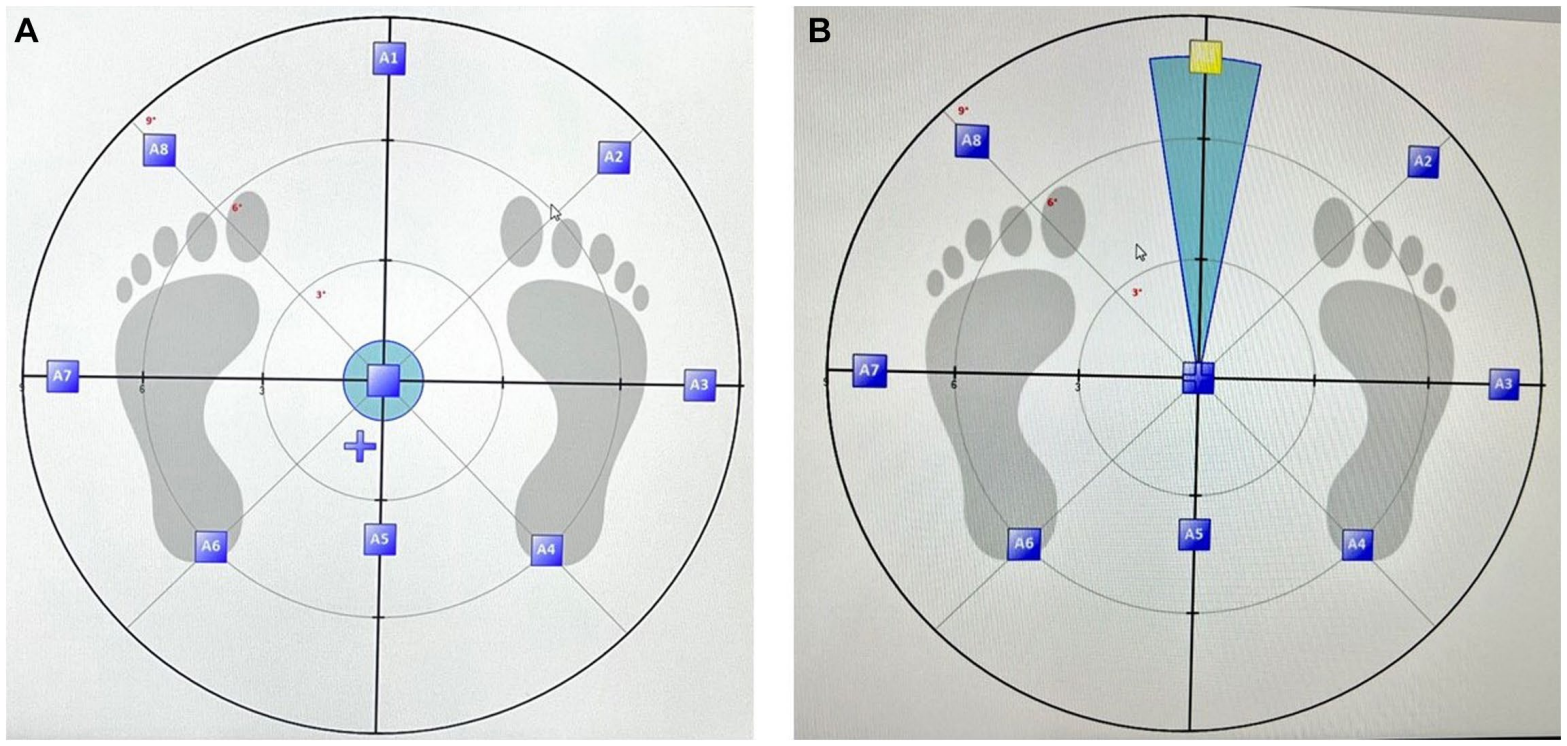
Assessment of Limits of Stability in eight directions, with **(A)** depicting the initial starting position and **(B)** demonstrating the participant’s reaching of a predefined target, as depicted in computerized posturography.

**Figure 2:**
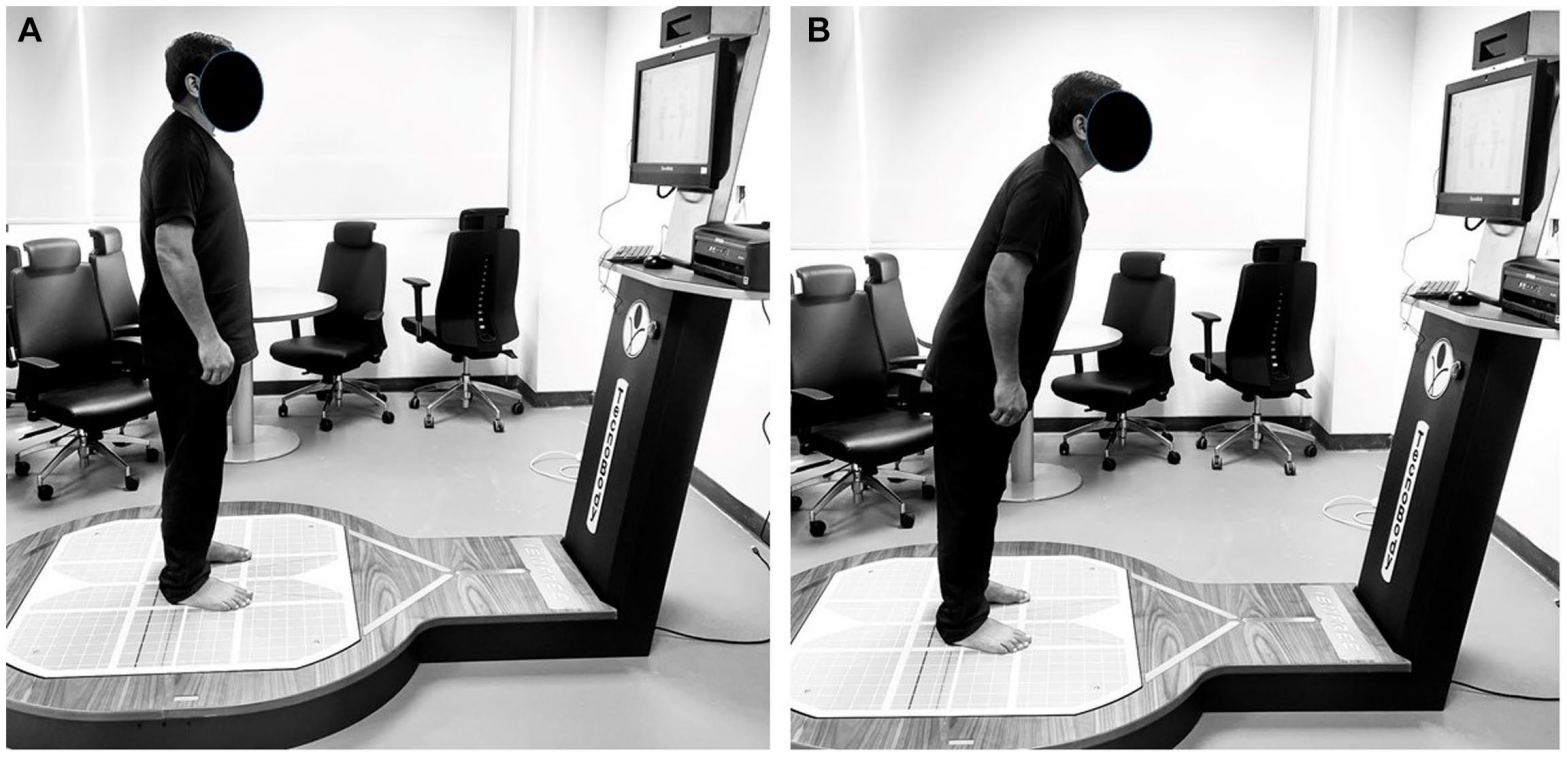
Evaluation of Limits of Stability, with **(A)** illustrating the initial starting position, and **(B)** illustrating the participant’s reaching of a determined target.

The force platform itself was calibrated and maintained according to manufacturer guidelines to ensure precise data collection (29). Each of the eight prescribed directions for shifting the COG was presented on a screen, providing a visual reference for participants during the assessment (Figures 1A,B). Additionally, a standardized protocol was implemented for the LOS assessment. Participants were instructed to perform the COG shifts with deliberate but controlled movements, ensuring that they did not lose balance or lift their heels off the platform (Figures 2A,B). The Isofree device randomized the eight testing directions, requiring participants to concentrate, reach each direction within a designated time frame, and subsequently return to a neutral position with guidance from computerized feedback. Participants were expected to execute this sequence consistently for all eight directions. This consistent approach across all participants contributed to the reliability of the assessment. To enhance the reliability of the LOS assessment, each subject completed the evaluation three times, with the best-performing trial selected for subsequent analysis. A one-minute rest period was incorporated between assessment sessions to mitigate potential fatigue effects.

#### 2.4.3 Timed up and go test

The TUG test served as a pivotal assessment tool in this study for evaluating participants’ functional mobility and balance (30). The TUG test is a well-established clinical assessment that quantifies the time required for an individual to transition from a seated position to a brief walking task (3 m/10 feet), encompassing a turn and a return to a seated position (31). This test offers a pertinent evaluation of an individual’s proficiency in executing fundamental functional movements and is frequently applied for the assessment of mobility and fall risk across diverse populations (31). Participants initiated the test from a seated position in a standard chair, and their performance was quantified in terms of the time taken to complete the entire TUG sequence. Subsequent analysis of the TUG test results revealed notable differences, with individuals afflicted by CLBP and osteoporosis exhibiting significantly prolonged TUG completion times in comparison to asymptomatic counterparts. This outcome implies compromised functional mobility and heightened susceptibility to falls among the former cohort (31). It furnishes invaluable insights into an individual’s capacity to execute essential everyday activities and is instrumental in guiding healthcare professionals in tailoring interventions to enhance mobility and diminish the risk of falls in individuals contending with conditions such as CLBP and osteoporosis (31).

#### 2.4.4 Berg balance scale test

The BBS was employed in this study to evaluate and quantify participants’ functional balance (32). This well-established assessment tool is widely used in clinical and research settings to gage an individual’s balance abilities (33). The BBS comprises a set of 14 balance-related tasks, each rated on a 5-point ordinal scale, ranging from 0 (unable to perform) to 4 (normal performance) (33). These tasks encompass a range of balance challenges, including sitting, standing, and dynamic movements such as reaching and turning (33). The total BBS score, with a maximum possible value of 56, serves as an overall measure of an individual’s functional balance, with higher scores indicating better balance abilities (33). The BBS was administered to participants to assess their functional balance. A trained evaluator observed and rated each participant’s performance on the 14 tasks, assigning scores based on their ability to complete the tasks safely and effectively. The intra-rater reliability of the BBS was found to be high, with a pooled estimate of 0.98 (95% CI 0.97 to 0.99). Similarly, relative inter-rater reliability also demonstrated high consistency, evidenced by a pooled estimate of 0.97 (95% CI 0.96 to 0.98) (34). The BBS is a valuable tool for assessing balance capabilities and fall risk in patients with various conditions, including chronic low back pain and osteoporosis.

### 2.5 Data analysis

The data analysis for this study was conducted using the Statistical Package for the Social Sciences (SPSS) software. Prior to analysis, the distribution of the data was examined, and it was determined that most of the data followed a normal distribution, satisfying the assumptions for parametric tests. Descriptive statistics were computed to summarize the demographic characteristics of the study cohort, including mean values and standard deviations for continuous variables. To assess the differences between individuals with osteoporosis and CLBP and asymptomatic individuals, independent t-tests were performed for continuous variables, Additionally, Pearson correlation coefficients (r) were calculated to assess the relationships between variables such as LOS tests, Functional Balance tests (TUG Score and BBS Score), and Kinesiophobia (TSK Score). Furthermore, mediation analyses were conducted to explore the mediating role of Kinesiophobia in the relationship between LOS tests and Functional Balance tests. The multiple linear regression analysis was employed to determine the presence of mediation, and Sobel tests were conducted to assess the significance of mediation effects. All statistical tests were two-tailed, and the significance level was set at *p* < 0.05 for all analyses.

## 3 Results

Table 1 summarizes the key findings of this study, which aimed to assess and compare various characteristics and clinical parameters within the study cohort, distinguishing between individuals concurrently experiencing osteoporosis and CLBP and asymptomatic counterparts. Notably, the two groups exhibited similar mean ages, with osteoporosis individuals with CLBP averaging 66.63 ± 6.67 years and asymptomatic individuals 65.99 ± 7.34 years (*p* = 0.345). However, it is essential to note that several clinical parameters, including BMI, pain intensity assessed through the Visual Analog Scale (VAS score), Oswestry Disability Index (ODI) score, Tampa Scale of Kinesiophobia (TSK Score), and Pain Catastrophizing Scale (PCS) score, did not apply to the asymptomatic cohort. Of significant clinical relevance, individuals with concurrent osteoporosis and CLBP displayed markedly lower T-scores in both lumbar and hip regions compared to their asymptomatic counterparts (*p* < 0.001), signifying a substantially elevated risk of osteoporosis-associated bone density loss in this population.

**Table 1:** Characteristics and clinical assessment parameters of the study cohort.

Parameters	Osteoporosis Individuals with CLBP (*n* = 86)	Asymptomatic individuals (*n* = 86)	*p*-value
Age, years	66.63 ± 6.67	65.99 ± 7.34	0.345
BMI, kg/m2	25.67 ± 3.47	24.78 ± 6.78	0.567
Pain intensity – VAS score (0–100 mm)	5.67 ± 1.34	–	–
ODI score (0–50)	33.45 ± 4.58	–	–
Kinesiophobia - TSK Score	29.95 ± 7.96	–	–
PCS score	23.56 ± 6.78	–	–
T-score (Lumbar)	−2.73 ± 1.19	0.21 ± 0.48	<0.001
T-score (hip)	−2.35 ± 0.86	0.65 ± 0.79	<0.001

BMI, Body Mass Index; VAS, Visual Analog Scale; ODI, Oswestry Disability Index; TSK, Tampa Scale of Kinesiophobia; PCS, Pain Catastrophizing Scale.

**Figure 3:**
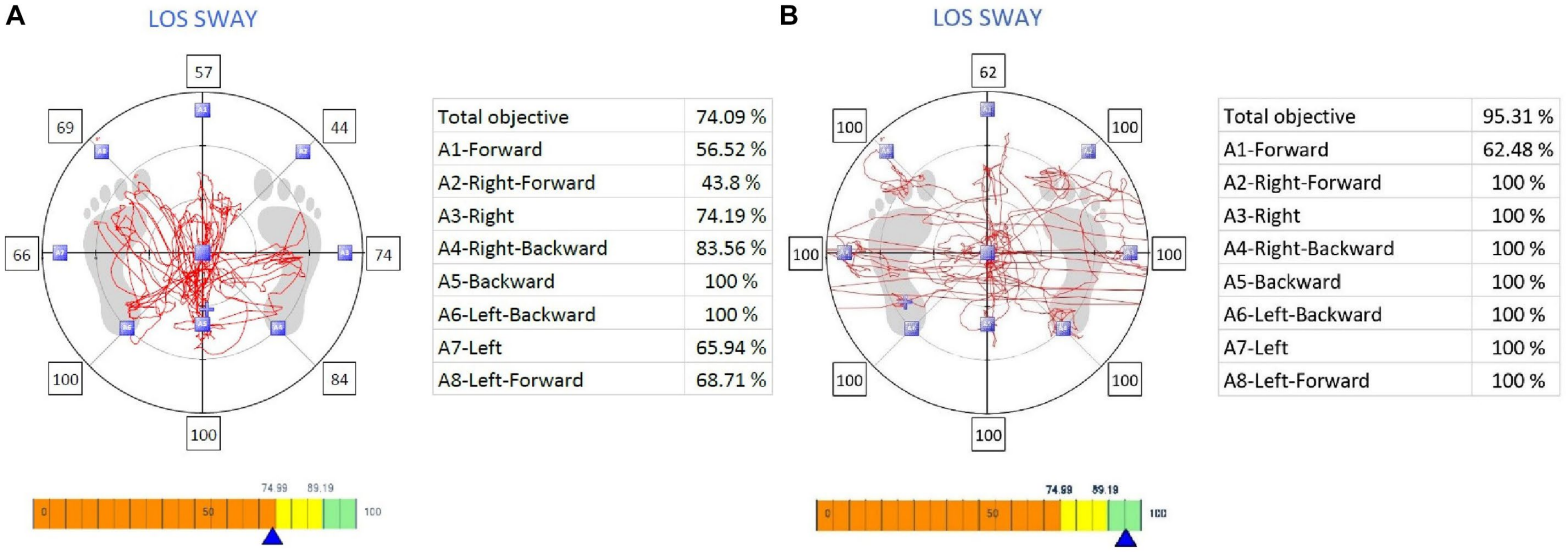
The Limits of Stability, with **(A)** representing individuals with Chronic Low Back Pain and osteoporosis, and **(B)** representing asymptomatic individuals for comparison.

The Table 2 and Figure 3 provides a comprehensive comparison of Limits of Stability and Functional Assessment Scores between two distinct groups: individuals with osteoporosis and CLBP and asymptomatic individuals. The results highlight significant differences in various parameters between these two groups, shedding light on the impact of osteoporosis and CLBP on physical function and stability. In terms of Limits of Stability (%), individuals with osteoporosis and CLBP demonstrated notably reduced scores in multiple directions compared to their asymptomatic counterparts (Figures 3A,B). Specifically, in the forward direction, the osteoporosis group scored significantly lower, with a mean of 40.18 ± 4.67, in contrast to the asymptomatic group’s score of 77.87 ± 8.97 (*p* < 0.001). Similar trends were observed in other directions, such as right-forward, right, right-backward, backward, left-backward, left, and left-forward. These findings collectively indicate that individuals with osteoporosis and CLBP exhibited compromised stability and balance, which is particularly evident in their reduced performance in various directional movements (all *p*-values <0.001).

**Table 2:** Comparison of limits of stability and functional assessment scores between osteoporosis individuals with CLBP and asymptomatic individuals.

Variable	Variable	Osteoporosis Individuals with CLBP (mean ± SD)	Asymptomatic individuals (mean ± SD)	*p*-value	*F*	Cohen’s *d*
Limits of stability (%)	Forward direction	40.18 ± 04.67	77.87 ± 08.97	<0.001	0.27	−3.32
	Right – Forward direction	67.89 ± 07.89	87.98 ± 10.87	<0.001	0.48	−1.86
	Right	71.05 ± 11.23	91.27 ± 11.23	<0.001	0.40	−1.78
	Right – Backward	88.88 ± 13.45	96.67 ± 13.56	<0.001	0.12	−0.59
	Backward	86.23 ± 12.22	94.24 ± 11.25	<0.001	0.20	−0.67
	Left – Backward	78.45 ± 09.98	89.97 ± 10.98	<0.001	0.53	−1.26
	Left	83.37 ± 09.78	93.67 ± 12.34	<0.001	0.26	−0.94
	Left – Forward	87.34 ± 11.23	96.89 ± 13.45	<0.001	0.21	−0.71
	Total objective	77.93 ± 09.87	95.67 ± 11.34	<0.001	0.48	−1.49
Functional balance tests (Score)	TUG score	10.45 ± 02.23	08.73 ± 01.90	<0.001	9.84	0.81
	BBS score	48.96 ± 05.23	55.32 ± 6.34	<0.001	6.34	−1.03

CLBP, Chronic low back pain; mean ± SD, mean ± SD (Standard Deviation); TUG score, Timed Up and Go Score; BBS score, Berg Balance Scale Score.

Functional Balance tests also revealed significant disparities between the two groups. For instance, the TUG Score, which assesses mobility and functional balance, yielded a mean score of 10.45 ± 2.23 for individuals with osteoporosis and CLBP, while asymptomatic individuals scored notably better with a mean of 8.73 ± 1.90 (*p* < 0.001). Additionally, the Berg Balance Scale (BBS) Score, a measure of balance and fall risk, showed lower scores among the osteoporosis group, with a mean of 48.96 ± 5.23, compared to the asymptomatic group’s mean of 55.32 ± 6.34 (p < 0.001).

Effect sizes, as indicated by Cohen’s d, underscored the magnitude of these differences. Negative Cohen’s d values across most parameters emphasized the poorer performance of individuals with osteoporosis and CLBP relative to asymptomatic individuals.

Table 3 provides valuable insights into the correlation between Limits of Stability tests and Functional Balance tests in patients grappling with both chronic low back pain (CLBP) and osteoporosis. The results of the correlation analysis are expressed through the Pearson correlation coefficient (r). A negative correlation signifies an inverse relationship, where reduced stability in a particular direction is associated with poorer functional balance, while a positive correlation indicates that greater stability is linked to improved functional balance.

**Table 3:** Correlation analysis of limits of stability and functional balance in patients with chronic low back pain and osteoporosis.

Parameters		Functional balance tests	
	Variables	TUG Score (*r*)	BBS score (*r*)
Limits of stability tests (%)	Forward direction	−0.378**	0.412**
	Right – Forward direction	−0.387**	0.489**
	Right	−0.414**	0.512**
	Right – Backward	−0.423**	0.532**
	Backward	−0.476**	0.541**
	Left – Backward	−0.389**	0.410**
	Left	−0.289**	0.423**
	Left – Forward	−0.313**	0.356**
	Total Objective	−0.387**	0.412**

“r”, Pearson correlation coefficient; TUG score, Timed Up and Go Score; BBS score, Berg Balance Scale Score, ** = statistically significant at *p* < 0.001.

The Forward direction in the LOS tests exhibited a notable negative correlation with both the TUG Score (r = −0.378, *p* < 0.001) and the BBS Score (r = 0.412, p < 0.001). This implies that individuals experiencing decreased stability when moving forward are more likely to exhibit compromised functional balance. Similarly, the Right-Forward direction in Limits of Stability demonstrated a negative correlation with the TUG Score (r = −0.387, p < 0.001) and a positive correlation with the BBS Score (r = 0.489, *p* < 0.001). These findings underscore the importance of stability in right-forward movements as a predictor of functional balance in patients with CLBP and osteoporosis.

Further analysis of different directional movements in Limits of Stability, including Right, Right - Backward, Backward, Left - Backward, Left, and Left-Forward, revealed consistently negative correlations with the TUG Score and positive correlations with the BBS Score, all of which were statistically significant (*p* < 0.001). This comprehensive pattern suggests that diminished stability in various directions is closely associated with compromised functional balance across the board. Additionally, the Total Objective score in Limits of Stability exhibited similar negative correlations with the TUG Score (r = −0.387, p < 0.001) and the BBS Score (r = 0.412, *p* < 0.001). This overarching finding emphasizes the overarching relationship between overall stability limitations and reduced functional balance in patients grappling with both CLBP and osteoporosis.

Table 4 presents the outcomes of a correlation analysis investigating the relationships among Kinesiophobia, Limits of Stability tests, and Functional Balance tests in patients concurrently experiencing CLBP and osteoporosis. A negative correlation was observed between Kinesiophobia and the Total Objective score in Limits of Stability tests (r = −0.362, *p* < 0.001), suggesting that individuals with higher Kinesiophobia levels tend to experience limitations in overall stability. Additionally, Kinesiophobia showed a negative correlation with the TUG Score (r = 0.322, p < 0.001), indicating that greater Kinesiophobia is linked to increased time required for tasks involving mobility and functional balance. Furthermore, a negative correlation was observed between Kinesiophobia and the BBS Score (r = −0.436, p < 0.001), indicating that heightened Kinesiophobia is associated with diminished performance on balance assessments, suggesting a detrimental impact on functional balance.

**Table 4:** Correlation analysis of Kinesiophobia, and limits of stability and functional balance in patients with chronic low back pain and osteoporosis.

Variables	Limits of stability tests (%)	Functional balance tests	
	Total objective score (*r*)	TUG Score (*r*)	BBS score (*r*)
Tampa scale of Kinesiophobia score – Kinesiophobia	−0.362**	0.322**	−0.436**

“r”, Pearson correlation coefficient; TUG score, Timed Up and Go Score; BBS score, Berg Balance Scale Score, ** = statistically significant at *p* < 0.001.

In our analysis, detailed in Table 5, we explored the relationships between Kinesiophobia (measured by the TSK score) and several key parameters, including Limits of Stability (LOS) and Functional Balance tests (TUG and BBS scores). The mediation analysis assessed the direct, indirect, and total effects of Kinesiophobia on these factors. Specifically, for the relationship between Kinesiophobia, LOS, and TUG score, a significant direct effect of Kinesiophobia was observed (B = 0.24, SE = 0.03, *p* < 0.001), indicating a direct relationship between Kinesiophobia levels and TUG test performance. Additionally, an indirect effect (B = 0.09, SE = 0.06, *p* = 0.002) and a total effect (B = 0.13, SE = 0.04, *p* = 0.001) were found, suggesting that Kinesiophobia influences TUG scores both directly and through its impact on LOS. Similarly, in the relationship between Kinesiophobia, LOS, and BBS score, there was a significant direct effect of Kinesiophobia (B = 0.38, SE = 0.03, p < 0.001), along with an indirect effect (B = 0.10, SE = 0.05, *p* = 0.003) and a total effect (B = 0.20, SE = 0.05, p = 0.002), highlighting the multifaceted impact of Kinesiophobia on balance as measured by the BBS score.

**Table 5:** Mediation analysis of Kinesiophobia between the relationship between limits of stability and functional balance tests.

Variable	Direct effect			Indirect effect			Total effect		
	*B*	*SE*	*p*-value	*B*	*SE*	*p*-value	*B*	*SE*	*p*-value
Kinesiophobia (TSK score) × LOS × TUG score	0.24	0.03	<0.001	0.09	0.06	0.002	0.13	0.04	0.001
Kinesiophobia (TSK score) × LOS × BBS score	0.38	0.03	<0.001	0.10	0.05	0.003	0.20	0.05	0.002

B, unstandardized coefficient; SE, Standard Error; TUG, Timed Up and Go score; BBS, Berg Balance Scale.

## 4 Discussion

This study pursued three primary objectives. Firstly, it aimed to evaluate the Limits of Stability and Functional Balance in patients simultaneously managing chronic low back pain (CLBP) and osteoporosis, comparing them to age-matched healthy controls. Secondly, it sought to establish correlations between Kinesiophobia, Limits of Stability, and Functional Balance within this specific patient population. Lastly, the study investigated the potential mediating role of Kinesiophobia in the relationship between Limits of Stability and functional balance among individuals with CLBP. The results underscore that patients with CLBP and osteoporosis exhibit reduced limits of stability and compromised functional balance. Notably, Kinesiophobia exhibited significant positive correlations with limits of stability and functional balance tests, and it also significantly mediated the relationship between limits of stability and functional balance parameters. These findings emphasize the substantial impact of Kinesiophobia on both stability and functional balance in these individuals, underscoring its significance for their physical well-being.

The observed reduction in LOS among patients with CLBP and osteoporosis suggests a compromised ability to control their center of mass and maintain balance during various movements (23). Several factors may contribute to this limitation. First and foremost, the pain associated with CLBP can disrupt neuromuscular control and affect postural stability (35). Chronic pain often leads to altered movement patterns and a reduced ability to make precise adjustments in posture and balance, ultimately affecting LOS (36). Additionally, individuals with osteoporosis face the added challenge of reduced bone density and strength, increasing their vulnerability to fractures and fall-related injuries, further impeding LOS (37). The compromised functional balance observed in this patient population is likely a multifactorial (38). Chronic low back pain itself can lead to functional impairments, limiting an individual’s ability to perform daily activities and maintain balance during tasks such as walking or standing (39, 40). Osteoporosis, characterized by weakened bone structure, also contributes to the risk of fractures, which can have a profound impact on functional balance (41). The fear of fractures due to osteoporosis may lead to a cautious and guarded approach to movement, potentially exacerbating functional balance limitations (42).

Several previous studies align with the observed reduction in LOS and functional balance among individuals with CLBP and osteoporosis (43–45). Soysal Tomruk et al. (43) research has shown that CLBP is associated with alterations in muscle activation patterns and proprioception, which can affect postural control and LOS. Additionally, studies examining the impact of osteoporosis on balance have highlighted the increased risk of falls and fractures in this population, underscoring the importance of addressing balance deficits (44). However, it is worth noting that some studies have explored interventions aimed at improving LOS and functional balance in older people (46, 47). These interventions often include targeted exercise programs that aim to enhance core stability, improve muscular strength, and promote proprioception (45, 48, 49). Implementing such interventions as part of a comprehensive management strategy may prove beneficial in mitigating the observed limitations in LOS and functional balance (45).

The consistent negative correlations observed in this study across various directional movements in LOS, such as forward, right, right-forward, right-backward, backward, left-backward, left, and left-forward, with the TUG Score and positive correlations with the BBS Score highlight the interdependence of stability and functional balance (50). These findings imply that individuals with reduced stability in different directions tend to exhibit compromised functional balance, emphasizing the clinical importance of addressing stability deficits in this cohort (51). Several factors contribute to these correlations. Chronic low back pain often disrupts neuromuscular control and proprioception, impacting an individual’s ability to make precise adjustments in posture and balance during various movements (52). This disruption can result in compromised stability, as evidenced by the negative correlations with LOS (52). Furthermore, osteoporosis, characterized by weakened bone structure, increases the risk of fractures and fall-related injuries, which can further impede stability and functional balance (53). These findings align with previous studies that have demonstrated the negative impact of CLBP and osteoporosis on postural control and balance, emphasizing the importance of comprehensive rehabilitation strategies to address these limitations (53). The negative correlation with the total objective score in LOS tests indicates that individuals with heightened kinesiophobia tend to experience limitations in their overall stability, while the negative correlation with the TUG Score suggests that greater kinesiophobia is linked to increased mobility-related time requirements. Most significantly, the negative correlation with the BBS Score emphasizes that elevated kinesiophobia is associated with diminished performance on balance assessments, indicative of a detrimental effect on functional balance (43). These findings collectively underscore the importance of addressing Kinesiophobia as a critical factor in the assessment and management of patients dealing with CLBP and osteoporosis, with implications for enhancing stability and functional outcomes (54).

Supporting the observed correlations, previous research has also highlighted the link between pain-related conditions and stability deficits (55). Studies on CLBP have shown that pain alters muscle activation patterns, affecting postural control and LOS (43, 56). Additionally, investigations into osteoporosis have emphasized the increased fall risk and balance impairments in individuals with reduced bone density (57). These studies collectively reinforce the significance of addressing both stability and functional balance in individuals managing CLBP and osteoporosis to mitigate the risk of falls, fractures, and compromised quality of life (43).

In the context of the relationship between Kinesiophobia, LOS, and functional balance, the findings suggest that Kinesiophobia significantly impacts an individual’s ability to control stability during various movements. This result is in line with previous research that has highlighted how fear of movement can lead to cautious and guarded behaviors, potentially altering neuromuscular control and proprioception, which are essential for maintaining stability (58). Additionally, the analysis reveals that Kinesiophobia operates through indirect mechanisms, further emphasizing its multifaceted influence on LOS and functional mobility (59). These findings underscore the importance of considering psychological aspects, such as Kinesiophobia, in the assessment and management of individuals with CLBP and osteoporosis, as they can significantly affect their stability and mobility.

### 4.1 Clinical significance and practical implications of the study

The clinical significance of our study is in informing more effective strategies for managing individuals with CLBP and osteoporosis. It elucidates the complex interplay between Kinesiophobia, stability, and functional balance, advocating for a comprehensive patient care approach. Utilizing these insights, healthcare providers can enhance care by integrating psychological support to mitigate movement fear and bolstering mobility confidence. Such a holistic strategy aims to reduce fall risk, elevate quality of life, and empower patient autonomy in physical health management. Key to this approach is the routine assessment of Kinesiophobia using tools like the Tampa Scale for Kinesiophobia to identify fear-avoidance behaviors. Early recognition of these behaviors enables the implementation of tailored interventions, including cognitive-behavioral therapy and educational support, thereby optimizing rehabilitation outcomes.

### 4.2 Limitations and future directions

While this study offers valuable insights into the relationship between Kinesiophobia, stability, and functional balance in individuals managing both CLBP and osteoporosis, it is not without limitations. The cross-sectional design limits the establishment of causality, and further longitudinal studies are warranted to elucidate the temporal relationships among these variables. Additionally, the study relied on self-reported measures for Kinesiophobia, which may introduce subjective bias. Future research should incorporate objective assessments of fear of movement and explore the potential role of other psychological factors. Moreover, a broader and more diverse patient population should be considered to enhance the generalizability of findings. Looking ahead, there is a critical need for future research to focus on developing and testing targeted interventions to reduce Kinesiophobia among patients with CLBP and osteoporosis. This could involve creating customized cognitive-behavioral therapy approaches or innovative physical therapy programs that specifically address fear-avoidance behaviors. Additionally, it is important to conduct longitudinal studies to assess the long-term effectiveness and sustainability of these interventions. Such research will not only fill existing knowledge gaps but also pave the way for more effective management strategies, ultimately enhancing the quality of life for this patient population. Finally, the study focused on correlations, and future investigations may benefit from interventional approaches to assess the effectiveness of targeted interventions in improving stability and functional balance in this patient population.

## 5 Conclusion and implications

In summary, our investigation elucidates the intricate interplay among Kinesiophobia, stability, and functional balance in the context of individuals concurrently experiencing CLBP and osteoporosis. These patients manifest compromised limits of stability and impaired functional balance. Significantly, our analysis unveils robust significant correlations between Kinesiophobia and assessments of both stability and functional balance, establishing Kinesiophobia as a salient mediating factor in this relationship. This underscores the imperative nature of integrating Kinesiophobia mitigation strategies within rehabilitation protocols to optimize personalized care, mitigate fall susceptibility, and enhance the overall well-being of these individuals. While further research is warranted to elucidate causality and design effective interventions, our findings provide valuable insights with the potential to reshape prevailing clinical methodologies for individuals grappling with the dual burden of CLBP and osteoporosis.

## Data availability statement

The raw data supporting the conclusions of this article will be made available by the authors, without undue reservation.

## Ethics statement

The research involving human subjects received ethical approval from the Ethics Research Board of King Khalid University (HAP0-06-B-001). The studies were carried out in adherence to local regulations and institutional mandates. Participants in the study provided written informed consent prior to their participation.

## Author contributions

MA: Conceptualization, Data curation, Formal analysis, Project administration, Resources, Software, Writing – original draft, Writing – review & editing. RR: Conceptualization, Data curation, Formal analysis, Funding acquisition, Methodology, Software, Writing – original draft, Writing – review & editing.
